# Cytotoxic NK cells phenotype and activated lymphocytes are the main characteristics of patients with alcohol-associated liver disease

**DOI:** 10.1007/s10238-023-01121-1

**Published:** 2023-07-01

**Authors:** Coral Zurera-Egea, Aina Teniente-Serra, Daniel Fuster, Eva Martínez-Cáceres, Roberto Muga, Paola Zuluaga

**Affiliations:** 1 Genetics of Male Fertility Group, Unitat de Biologia Cel·lular (Facultat de Biociències), Departament de Biologia Cel·lular Fisiologia i Immunologia, Universitat Autònoma de Barcelona, Cerdanyola del Vallès, Spain; 2 Department of Inmunology, Hospital Universitari Germans Trias I Pujol, IGTP, Badalona, Barcelona Spain; 3 Universitat Autònoma de Barcelona, Barcelona, Spain; 4 Department of Internal Medicine, Hospital Universitari Germans Trias I Pujol, IGTP, Ctra. Canyet S/N, 08916 Badalona, Barcelona Spain

**Keywords:** Cytotoxic NK cells, Activated T cells, NKT-like, Alcohol-associated liver fibrosis

## Abstract

T cells, natural killer (NK) and NKT cells have opposing actions in the development of alcohol-associated liver fibrosis. We aimed to evaluate the phenotype of NK cells, NKT cells and activated T cells in patients with alcohol use disorder (AUD) according to the presence of advanced liver fibrosis (ALF). Totally, 79 patients (51-years, 71% males) were admitted to treatment of AUD. ALF was defined as FIB4-score > 2.67. Immunophenotyping of NK cells (CD3^−^CD56^+^CD16^+^, CD3^−^CD56^+^CD16^−^, CD3^−^CD56^−^CD16^+^), NKT-like (CD3^+^CD56^+^), and the activation status of CD4^+^, CD8^+^ and regulatory T cells (Tregs) were evaluated according to the HLA-DR expression. Patients had an AUD duration of 18 ± 11 years with a daily alcohol consumption of 155 ± 77 gr/day prior to hospital admission. The values of absolute cells were 2 ± 0.9 cells/L for total lymphocytes, 1054 ± 501 cells/µL for CD4^+^, 540 ± 335 cells/µL for CD8^+^, 49.3 ± 24.8 cells/µL for Tregs, 150.3 ± 97.5 cells/µL for NK cells and 69.8 ± 78.3 cells/µL for NKT-like. The percentage of total NK cells (11.3 ± 5.5% vs. 7 ± 4.3%, *p* < 0.01), CD3^−^CD56^+^CD16^+^ regarding total lymphocytes (9.7 ± 5.1% vs. 5.8 ± 3.9%, *p* < 0.01), activated CD4^+^ cells (5.2 ± 3.2% vs. 3.9 ± 3%, *p* = 0.04) and activated CD8^+^ cells (15.7 ± 9.1% vs. 12.2 ± 9%, *p* = 0.05) were significantly higher in patients with ALF. The percentage of CD3^−^CD56^+^CD16^−^ regarding NK cells (5.1 ± 3.4% vs. 7.6 ± 6.2%, *p* = 0.03) was significantly lower in patients with ALF. Activated Tregs (39.9 ± 11.5 vs. 32.4 ± 9.2, p = 0.06) showed a tendency to be higher in patients with ALF. The proportion of activated CD4^+^ cells (*r* = 0.40, *p* < 0.01) and activated CD8^+^ cells (*r* = 0.51, *p* < 0.01) was correlated with the proportion of NKT-like in patients without ALF. Patients with ALF presented an increased NK cytotoxic phenotype and activated T cells concomitant with a decreased NK cytokine-secreting phenotype.

## Introduction

Alcohol-associated liver disease (ALD) is the leading cause of death from liver disease in the world. The presence of liver fibrosis is the most relevant predictive factor of morbidity and mortality in patients with ALD [1].

Hepatic stellate cell (HSC) activation is the main mediator in the formation and progression of liver fibrosis. Immune responses and hepatic inflammation are triggering factors for HSC activation [2].

NK cells are believed to be involved in fibrosis prevention through the inhibition of HSC activation [3, 4]. Particularly, NK cells can operate as cells able to specifically kill senescent HSC and able to induce cell cycle arrest and apoptosis in HSC [5, 6]. NK cell interactions with HSCs and their activity are regulated by various immune cell types, such as dendritic cells, Kupffer cells and T cells [7, 8]. This dysregulated activity of NK cells may promote healthy hepatocyte apoptosis through the same mechanisms already studied for HSC and has been associated with the progression of ALD [3, 9].

NK cells are divided into several subtypes depending on expression of CD16 and CD56 proteins [10]. Expression of these markers is transitory and related to NK cell maturation. Three NK cell subtypes are defined in peripheral blood: immunoregulatory/immature CD56^bright^CD16^−^ NK cells (also called CD56^bright^), cytotoxic/mature CD56^dim^CD16^+^ NK cells, which are the most common, and the less mentioned memory-like CD56^−^CD16^+^ NK cells, which are the ending stage of maturation [11, 12]. Opposite to what is seen in peripheral blood, approximately half of the liver NK cells present the CD56^bright^ phenotype [13, 14]. However, CD56^bright^ tissue-resident liver NK cells seem to have an increased activation pattern with increased CD69, granzyme and perforin expression, and increased chemokine and cytokine receptors expression. They also have a differential functionality to the peripheral blood subsets as they are unable to produce Th2-like cytokines, but instead have cytolytic capacities by granzyme degranulation. Those previously mentioned traits significantly differentiate CD56^bright^ liver NK cells compared to their peripheral blood counterparts [14].

NKT cells are a subset of unconventional T cells that express the receptor of T cells (TCR) and receptors of NK cells. They have immunoregulatory activity and are divided into two different subtypes [8, 15]. Type I NKT cells have been observed to increase after ethanol consumption while their type II counterparts are not affected [16]. The role of NKT cells on ALD is controversial, as well as their definition by surface markers. While NKT I cells are defined by their expression of an invariant TCR or the surface protein CD1d, some studies define them as NKT-like cells by the coexpression of CD3 with CD56 markers [17]. Although NKT cells have been reported to kill activated HSC, it should be noted that a subset of these cells can release IL-4, IL-13 and Hedgehog ligands as well to promote HSC activation and liver fibrosis [18].

Tregs are a specific T cell subset from the adaptive immune system which in homeostasis is involved in the suppression of excessive immune responses that might lead to healthy tissue damage [19]. In the context of liver disease, Tregs are believed to suppress the activity of NK cells and to enhance liver fibrosis progression through IL-8 release [20]. Tregs have also been observed to highly suppress CD56^bright^ NK cells activity while not affecting CD56^dim^ NK cells under the same conditions [7].

Moreover, regarding conventional T cells, both CD4^+^ (helper phenotype) and CD8^+^ (cytotoxic phenotype) T cells have been observed in previous studies to increase in AUD patients, which have increased liver damage parameters, compared to healthy controls [21]. Furthermore, T cell dysfunction and activation have been associated with liver fibrosis onset and progression in other contexts, such as non-alcoholic steatohepatitis [22].

Overall, the immune system participates in shaping the pathogenesis of the alcohol-related liver disease [23]. We hypothesize that the peripheral immune cell profile and cellular interactions may differ between patients with and without advanced liver fibrosis. This study aimed to evaluate, through flow cytometry, the phenotype of NK cells, NKT-like cells and the activation profile of T cells in AUD patients according to the presence or absence of advanced liver fibrosis. In addition, we aimed to determine correlations of NK cells and NKT-like cells with the T cell activation patterns.

## Materials and methods

### Patients: admission, exclusion, and stratification criteria

This was a cross-sectional study including 79 patients admitted for alcohol use disorder (AUD) treatment in Hospital Germans Trias i Pujol, Badalona, Spain. This study was conducted between April 2019 and July 2020. These patients were diagnosed with AUD according to the Diagnostic Statistical Manual of Mental Disorders (DSM-5) criteria [24]. The main criteria considered for hospital admission were severity of AUD, risk of severe withdrawal syndrome and difficulty in following outpatient treatment. On the first day of admission, medical history of alcohol consumption (age at drinking onset, quantity consumed in grams per day and duration of the disorder) and consumption of other substances in the last month were considered and noted down.

**Fig. 1:**
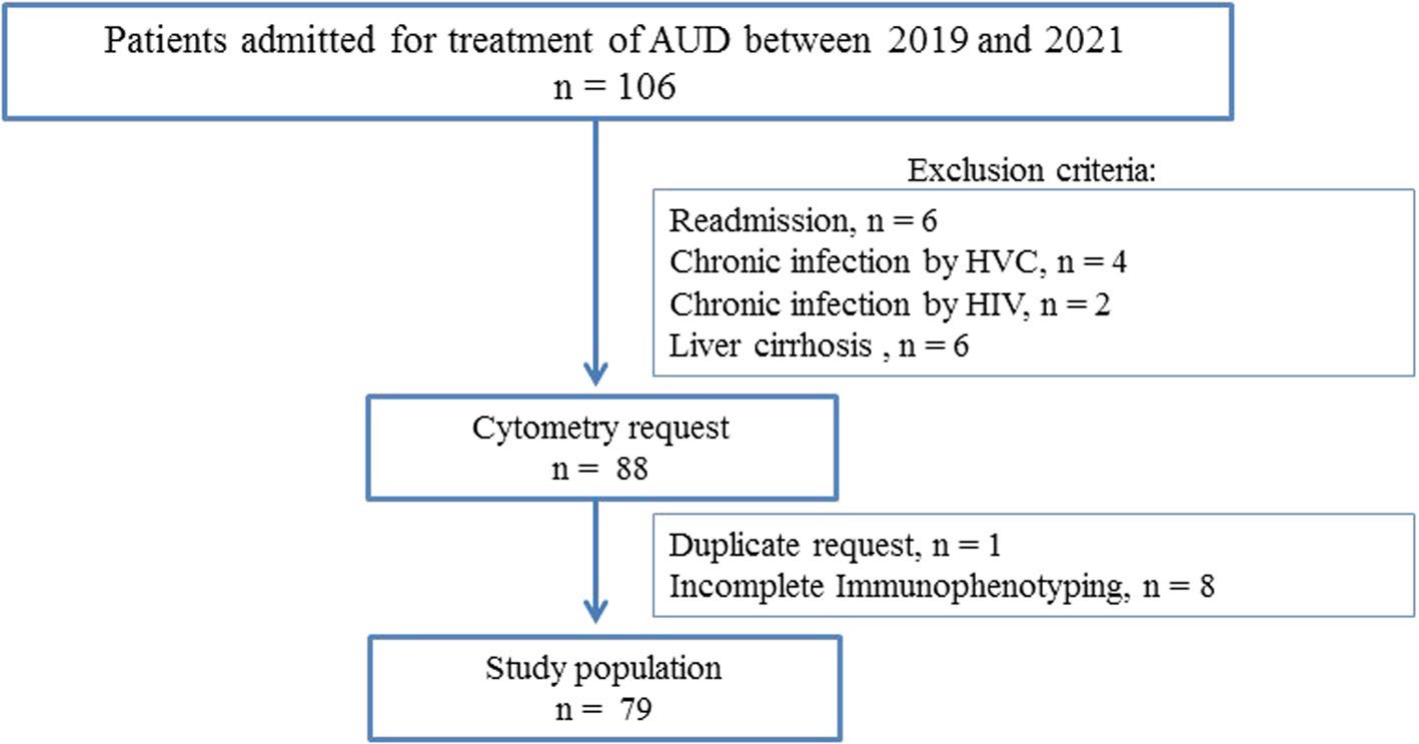
Study flowchart. The figure shows the exclusion criteria and study population

On the second day of admission, we compiled the baseline laboratory parameters, such as whole blood cell count, liver panel and chronic hepatitis C virus infection (HCV), human immunodeficiency virus (HIV) and chronic hepatitis B virus infection (HBV) serology status. Serological tests were performed using an enzyme-linked immunosorbent assay (ELISA). HCV-positive tests were further confirmed using HCV rtPCR (real-time polymerase chain reaction, limit of detection 50 copies/mL). HIV-positive tests were confirmed using the Western immunoblot technique, and HBV serostatus was assessed using HBsAg, anti-HBs and anti-HBc. To analyse patients with autoimmune hepatitis, we tested antinuclear antibodies. The presence of advanced liver fibrosis was estimated by the FIB-4 index. We dichotomized our study into 2 groups based on the FIB-4 index cut-off at 2.67 [25]. We excluded readmission and patients with HCV, HIV, HBV infection, autoimmune hepatitis or history of liver cirrhosis (Fig. 1).

### Flow cytometry whole-blood immunophenotyping

Peripheral whole-blood immunophenotyping of included patients was also performed during the second day of admission.

Immunophenotyping of peripheral whole blood studying NK cells, NKT-like cells and T cells was performed through multiparameter flow cytometry. After venepuncture, fresh whole blood of the study subjects was aliquoted in 100 µL amounts into polystyrene/polypropylene tubes and incubated for 20 min with different mixes of fluorochrome-conjugated monoclonal antibodies at room temperature and away from light exposure.

After incubation period, stained samples were subjected to erythrocyte lysis using 2 ml of BD-FACS lysing solution (Beckton Dickinson (BD) Biosciences, CA, USA) for 7 min. Samples were then washed and resuspended in flow cytometry staining buffer (BD-FACSFlow, BD Biosciences) for subsequent cytometer acquisition (LSR Fortessa and FACSCanto (BD Biosciences, San Jose, CA, USA)).

Cell subpopulations were studied using combinations of the following fluorochrome-conjugated monoclonal antibodies: anti-CD4 PerCPCy5.5, anti-CD25 PE, anti-CCR4 PE-Cy7, anti-CD127 AlexaFluor647, anti-CD45 AlexaFluor700, anti-CD45RO APC-H7, anti-CD3 BrilliantViolet450, anti-HLA-DR BrilliantViolet500, anti-CD45 FITC, anti-CD56 PE, anti-CD16 APC, anti-CD8 APC-H7, anti-CD19 BrilliantViolet500.

Absolute counts for each cell subset were calculated as (X x Y)/100, where X is the percentage of each subset and Y the absolute lymphocyte count determined using an haematology analyser (Beckman Coulter, FL, USA). Absolute numbers and percentages of total lymphocytes (CD45^+^), T cells (CD3^+^), NKT-like cells (CD3^+^CD56^+^), cytotoxic CD56^+^CD16^+^, cytokine-secreting CD56^+^CD16^−^ and memory-like CD3^−^CD56^−^CD16^+^NK cells, and activated subsets of CD8^+^ T cells, CD4^+^ T cells and Tregs based on HLA-DR expression, were obtained. Absolute numbers obtained were expressed in cells per microliter (cells/µL).

Regarding the gating strategy of lymphocyte subsets obtained through flow cytometry, doublet cells were excluded, followed by the gating of lymphocytes based on their granularity and size characteristics also subsequently based on their CD45^+^ expression. Following said gating, several subsets were defined based on their expression of lineage markers CD3^+^, CD4^+^ and CD8^+^.

### Statistical analysis

Results of the descriptive analysis are expressed as means ± standard deviation (SD) or median (interquartile range (IQR)) and as absolute frequencies and percentages for qualitative variables. To analyse the differences between the means of both patients’ groups (ALF and non-ALF), a T-test or a U–Mann–Whitney nonparametric test was used according to the distribution of each variable. To analyse bivariate correlations between cell subsets, Spearman’s Rho test was used. Statistical significance was set at *p* < 0.05. Statistical analyses were performed through the SPSS software (IBM SPSS Statistics 23.0).

## Results

### Baseline demographic, laboratory parameters and absolute values of T cells

The mean age of our study cohort was 51 years; most patients were male (71%). Patients had a mean age at starting alcohol consumption of 16 ± 3 years and a mean AUD duration of 18 ± 11 years with a daily alcohol consumption of 155 ± 77 gr/day. The mean of leukocytes, lymphocytes, haemoglobin, platelets and erythrosedimentation rate (ESR) among our patients was 6.32 ± 1.86 cells/µL, 2.08 ± 0.9 cells/µL, 13.48 ± 1.99 g/L, 209.81 ± 87.53 cells/µL and 27.23 ± 27.56 mm, respectively.

According to the FIB-4 index, we found that 18 (22.8%) AUD patients had advanced fibrosis. Table 1 describes the baseline and alcohol consumption characteristics according to the degree of liver fibrosis. Patients with advanced fibrosis had a lower haemoglobin level (11.7 ± 2.3 vs. 14 ± 1.55, *p* < 0.01), a lower platelets count (108.2 ± 42.2 vs 239.7 ± 73.7, *p* < 0.01) and a lower absolute leukocytes count (5 ± 2 vs. 6.7 ± 1.5, *p* < 0.01). The proportion of lymphocytes was lower (26.6 ± 11 vs. 34.6 ± 10.6, *p* < 0.01) and the proportion of neutrophils was higher (59.9 ± 10.8 vs. 51.8 ± 10.4, *p* < 0.01) in patients with advanced fibrosis. The proportion of eosinophils (3.5 ± 1.8 vs. 3.1 ± 2.1, *p* = 0.29) and monocytes (9 ± 1.8 vs. 9.3 ± 2.8, *p* = 0.97) was similar in the two groups.

**Table 1:** Baseline haematological parameters according to the presence of advanced liver fibrosis

Variables (means ± SD)	Non advanced liver fibrosis (*n* = 61)	Advanced fibrosis (*n* = 18)	*p*-Value
Years	47 ± 9.6	55.33 ± 9.24	< 0.01
Males (%)	42 (68.9)	14 (77.8)	–
Daily Alcohol Consumption (g/day)	150 ± 70.76	173.06 ± 96.36	0.489
Starting Alcohol Consumption (years)	16.9 ± 3.39	15.67 ± 3.29	< 0.01
AUD duration (years)	15.41 ± 9.81	26.95 ± 12.17	< 0.01
FIB-4 index	1.23 ± 0.54	6.48 ± 4.24	< 0.01

**Fig. 2:**
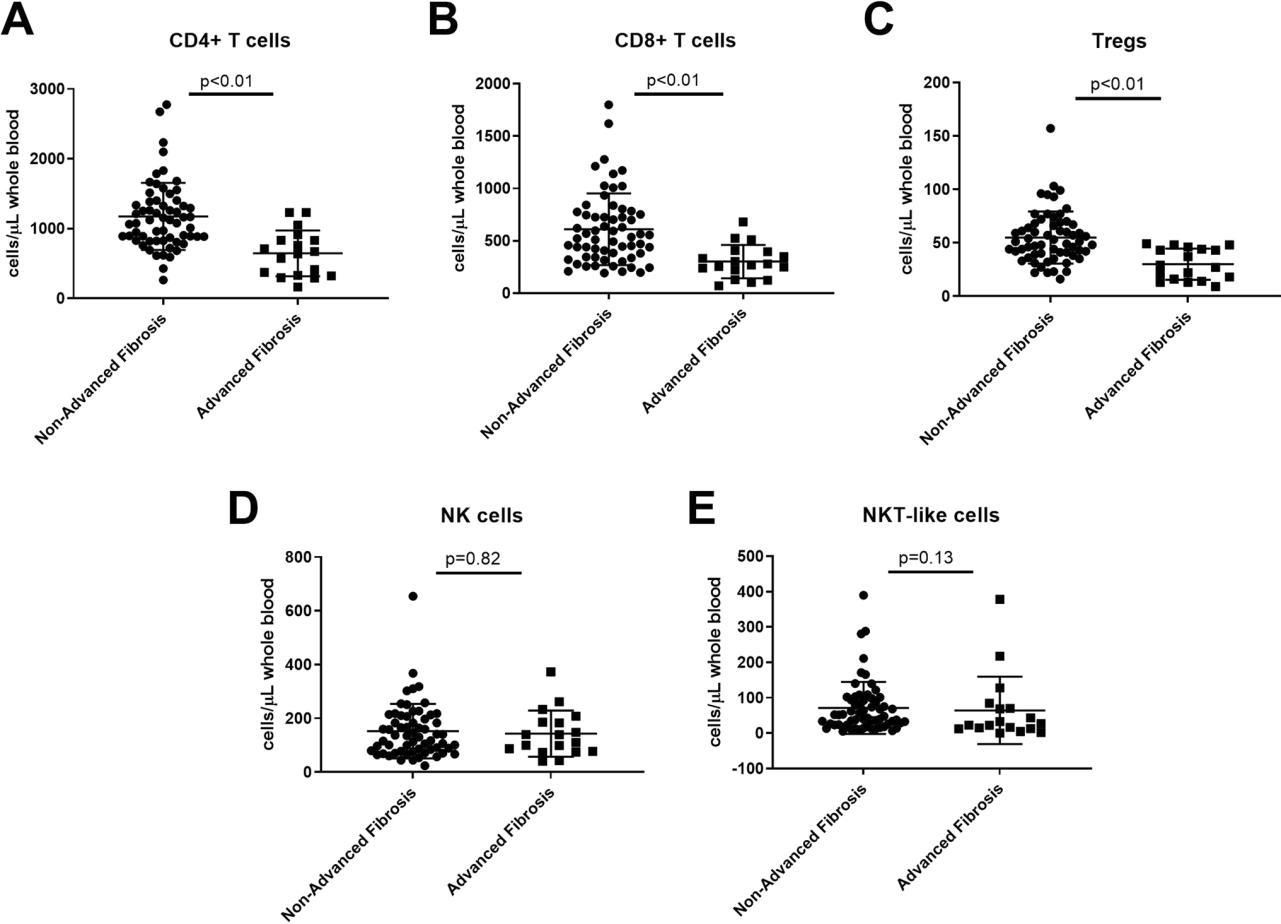
Differences observed in absolute values (cells/uL) of CD4 + T cells (646.66 ± 327.68), CD8 + T cells (302.25 ± 159.18), Tregs (30 ± 14.59) total NK cells (143 ± 85.75) and NKTs (64.35 ± 95.28) in ALF patients compared to CD4 + T cells (1174.87 ± 481.87), CD8 + T cells (610.05 ± 342.64), Tregs (54.97 ± 24.35), total NK cells (152.48 ± 101.22) and NKTs (71.41 ± 73.42) in non-ALF patients, according to FIB-4

Absolute values of CD4^+^ T cells, CD8^+^ T cells and Tregs were significantly decreased in patients with advanced fibrosis (*p* < 0.01). However, no significant differences were observed between groups when evaluating absolute values of NK and NKT-like cells (Fig. 2).

### NK cell subsets and NKT-like cells regarding total lymphocytes

The percentage of total NK cells (11.3 ± 5.5 vs. 7 ± 4.3, *p* < 0.01) and the cytotoxic CD56^+^CD16^+^ NK cell (9.7 ± 5.1 vs. 5.8 ± 3.9, *p* < 0.01) were significantly higher in patients with advanced fibrosis. No differences were observed in CD56^+^CD16^−^ NK cells (0.5 ± 0.4 vs. 0.5 ± 0.4, *p* = 0.45) nor in CD56^−^CD16^+^ NK cells (1.2 ± 1.6 vs. 0.7 ± 0.9, *p* = 0.169) between groups. The percentage of total NKT-like cells (6 ± 8.7 vs. 3.3 ± 3.6, *p* = 0.73) was also similar in patients with and without advanced fibrosis.

### NK cell subsets regarding total NK cells

**Fig. 3:**
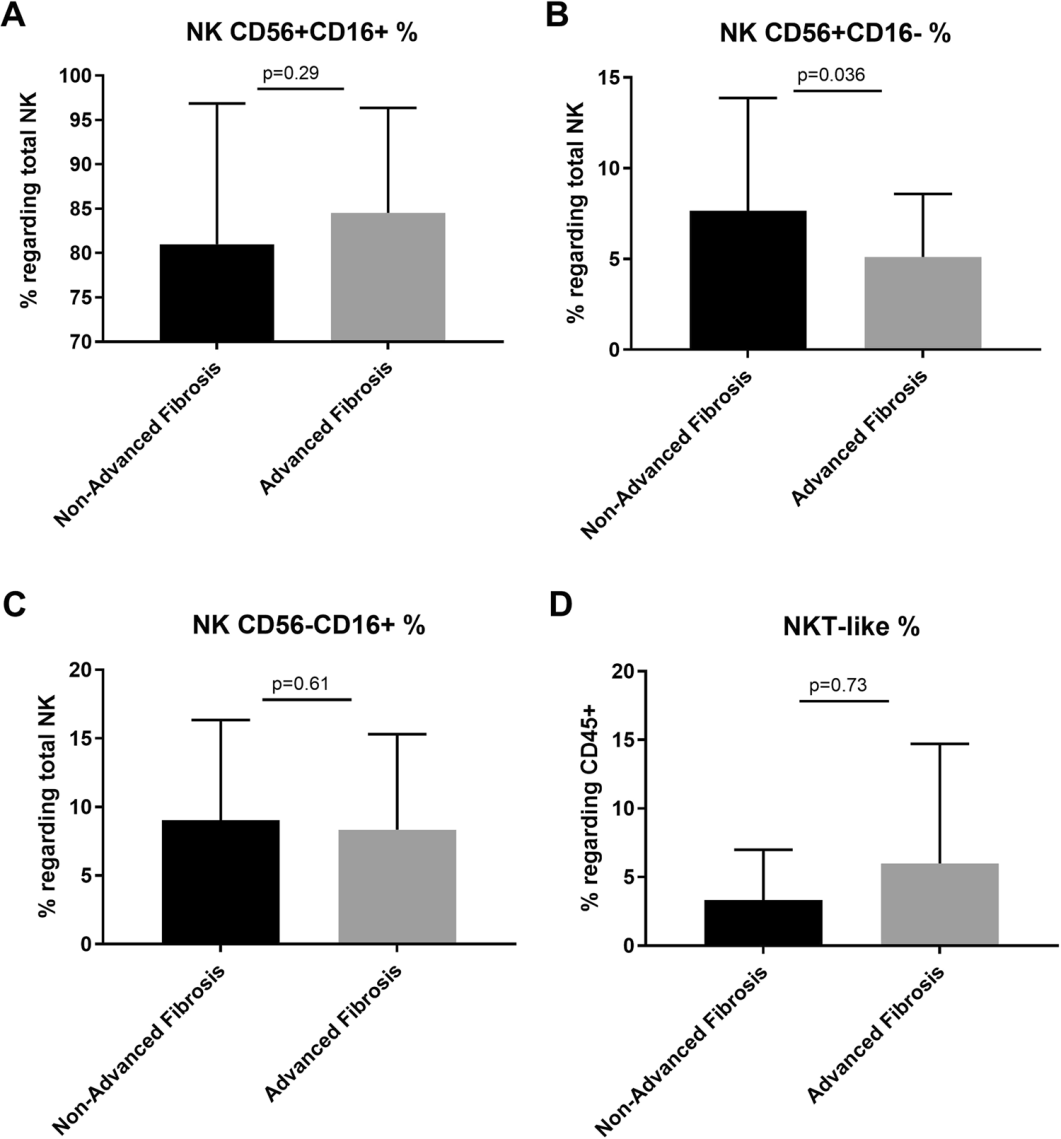
Differences in percentages (%) of CD56^+^CD16^−^NK cells (5.1 ± 3.47), CD56^+^CD16^+^NK cells (84.54 ± 11.82), CD56^−^CD16^+^ NK cells (8.34 ± 6.97) regarding total NK cells and NKT-like cells (6 ± 8.71) regarding total lymphocytes in ALF patients compared to CD56^+^CD16^−^NK cells (7.65 ± 6.22), CD56^+^CD16^+^NK cells (81 ± 15.87), CD56^−^CD16^+^NK cells (9.05 ± 7.29), and NKT-like cells (3.33 ± 3.67; 3.37 ± 3.74) in non-ALF patients, according to FIB-4

While the percentage of cytotoxic CD56^+^CD16^+^ NK cells was higher in patients with advanced fibrosis though not statistically significant (84.5 ± 11.8 vs. 81 ± 15.8, *p* = 0.29), the percentage of immature cytokine-secreting CD56^+^CD16^−^ NK cell was significantly lower in patients with advanced fibrosis (5.1 ± 3.4 vs. 7.6 ± 6.2, *p* = 0.03). The percentage of CD56^−^CD16^+^ NK cell was similar in both groups (8.3 ± 6.9 vs. 9 ± 7.2, *p* = 0.60) (Fig. 3).

### Activated T cells

**Fig. 4:**
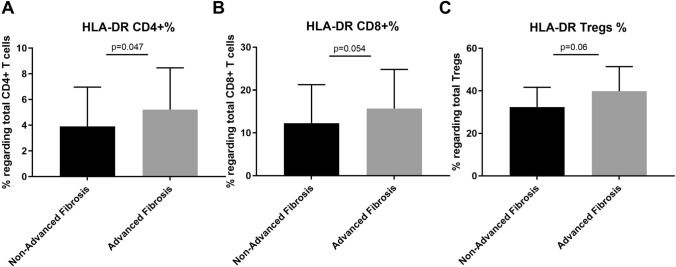
Differences in percentages (%) of HLA-DR-expressing cells in CD4^+^ T cells (5.22 ± 3.25), CD8^+^ T cells (15.69 ± 9.11), and Tregs (39.86 ± 11.55) in ALF patients compared to HLA-DR-expressing cells in CD4^+^ T cells (3.9 ± 3.07), CD8^+^ T cells (12.26 ± 9.01), and Tregs (32.45 ± 9.25) in non-ALF patients, according to FIB-4

The expression of HLA-DR in CD4^+^ T cells (5.2 ± 3.2 vs. 3.9 ± 3, *p* = 0.04) and CD8^+^ T cells (15.7 ± 9.1 vs. 12.2 ± 9, *p* = 0.05) was significantly higher in those patients with advanced fibrosis. The expression of HLA-DR in Tregs (39.9 ± 11.5 vs. 32.4 ± 9.2, *p* = 0.06) showed a tendency to be higher in those patients with advanced fibrosis (Fig. 4).

### Correlation between NK subsets and NKT-like cell with activated T Cells.

**Fig. 5:**
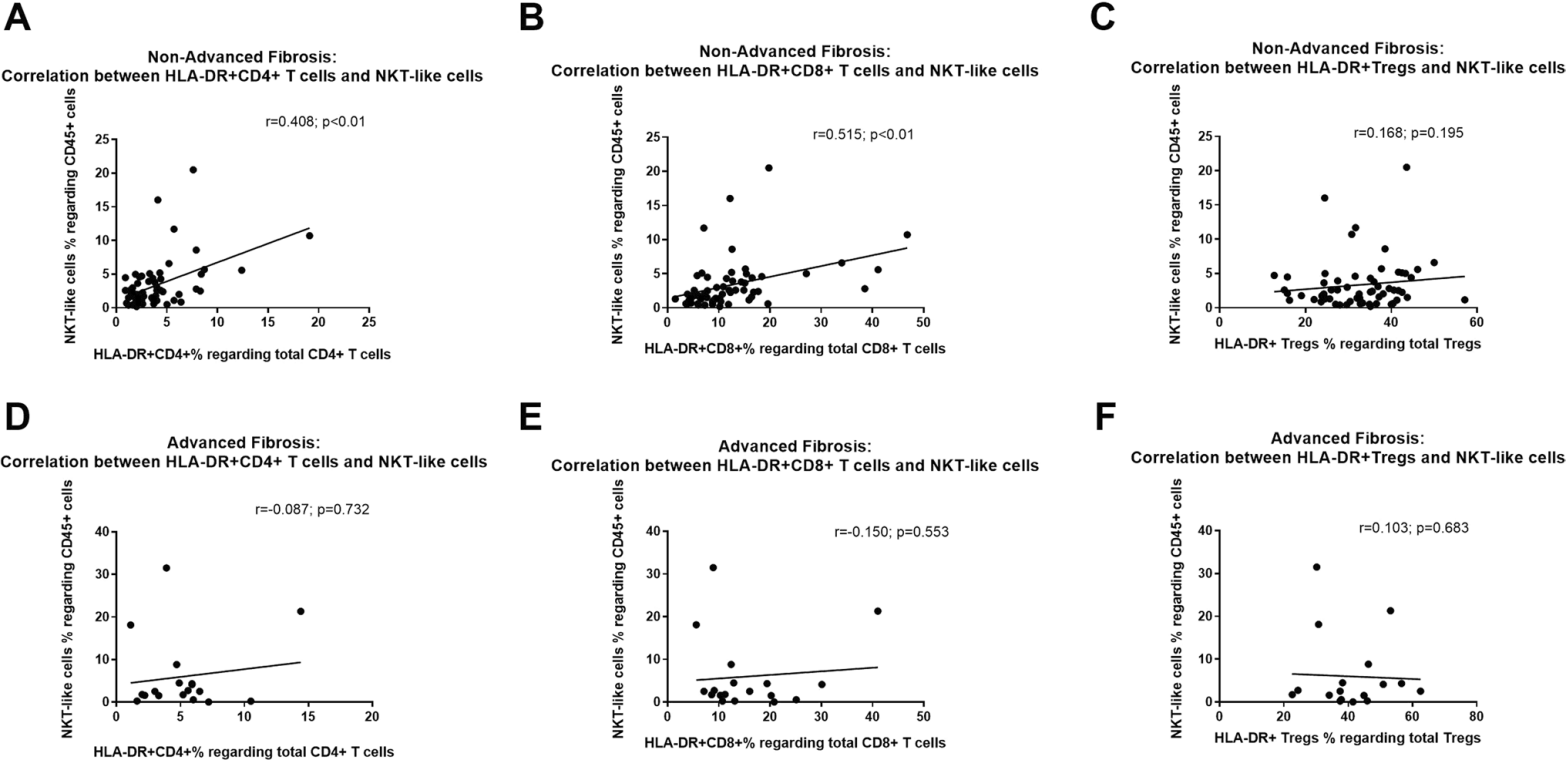
Correlations between HLA-DR-expressing CD4^+^ and NKT-like cells (*p* < 0.01; *r* = 0.41) and CD8^+^ T cells and NKT-like cells (*p* < 0.01; *r* = 0.52) in non-advanced liver fibrosis patients

There was no correlation between the percentage of HLA-DR-expressing CD4^+^ T cells, HLA-DR-expressing CD8^+^ T cells or HLA-DR-expressing Treg cells with the percentage of cytotoxic CD56^+^CD16^+^ NK cell nor immature cytokine-secreting CD56^+^CD16^−^ NK cells in any group. Also, there was no correlation between the percentage of HLA-DR-expressing CD4^+^ T cells, HLA-DR-expressing CD8^+^ T cells or HLA-DR-expressing Treg cells with the percentage of NKT-like cells in patients with advanced fibrosis. However, the proportion of HLA-DR-expressing CD4^+^ T cells (*r* = 0.40, *p* ≤ 0.01) and HLA-DR-expressing CD8^+^ T cells (*r* = 0.51, *p* ≤ 0.01) was correlated with NKT-like cells in patients without advanced fibrosis. There was no correlation between HLA-DR-expressing Treg cells and NKT-like (*r* = 0.1, *p* = 0.19) in those patients (Fig. 5).

## Discussion

The results of this study suggest that AUD patients with advanced liver fibrosis have more cytotoxic CD56^+^CD16^+^ NK cells, fewer cytokine-secreting CD56^+^CD16^−^ NK cells and an increased lymphocyte activation, defined by HLA-DR expression, than patients without advanced liver fibrosis. Our group previously described the increase in the absolute counts of NK cells and cytotoxic T cells subsets in patients with advanced fibrosis, but these results expand the description of the phenotype of NK [26].

In addition to the decrease in absolute counts of lymphocytes, the proportion of neutrophils was significantly increased in those patients. The increase in the neutrophil-to-lymphocyte ratio has been described in cancer research to be related to a poor prognosis and increased risk of hepatocarcinoma progression, and it is defined as a marker of poor prognosis in other diseases [27, 28]. In this context, this could indicate worse liver function or increased risk of liver disease progression, as the infiltration of neutrophils in the liver associated with an increased inflammatory status and tissue damage [29–31].

The proportion of total NK cells regarding lymphocytes was higher in patients with advanced fibrosis. The increase and activation of NK cells are the result of proinflammatory signals, especially IFNγ, coming from Th1 cells and active dendritic cells. However, our results did not demonstrate the correlation between the active T cells and the NK cells in either of the two groups. In patients with advanced liver fibrosis, we observed an increasing proportion of cytotoxic CD56^+^CD16^+^ NK cells regarding the whole lymphocyte count and decreased proportion of the cytokine-secreting CD56^+^CD16^−^ NK cell subset regarding total NK cells. These findings suggest that the increased proportion of NK cells could be at the expense of cytotoxic phenotype. It is know that chronic alcohol exposure attenuates the antifibrotic activity of NK cells, promoting resistance to apoptosis in senescent HSCs [32]. In this context, overactivation and the cytotoxic phenotype of NK cells could be related to tissue damage rather than to the neutralization of HSCs. In fact, the increased maturation of NK cells into the cytotoxic phenotype seems to be characteristic of advanced stages of liver fibrosis [33]_._

NKT cells are another subset that has been described with the development of ALD. Ethanol consumption is believed to induce type I NKT cells activity, which is involved with neutrophil recruitment and inflammation related to tissue damage, as previously mentioned. However, ethanol consumption does not affect type II NKT cells which are presumably related to beneficial effects on ALD [16]. In our study, we could not detect whether NKT cells were affected, and the lack of results in this regard might be due to the lack of markers differentiating the main two subtypes, although the positive correlation between activated CD4^+^ and CD8^+^ T cells and NKT in patients without advanced fibrosis suggests that their function is linked to T lymphocyte activation and therefore to a T Helper response. This correlation might indicate a possible feedback mechanism that favours the antifibrotic response, feedback that does not occur when liver fibrosis is already stablished. Therefore, these correlations could be furtherly studied and could be measured through co-culturing, evaluating the type of NKT cell responses while interacting with activated T cells.

Conventional T cell activation has also been reported in previous studies by the increased expression of HLA-DR and CD38 on CD4^+^ and CD8^+^ T cell subsets [10, 21]. In the current study, we used the main histocompatibility complex protein HLA-DR to determine cellular activation in both CD4^+^ and CD8^+^ T cells, and in Tregs. We observed that, as previously reported, activated CD4^+^ cells, CD8^+^ T cells are increased in patients with advanced fibrosis compared to patients without liver fibrosis [21, 34].

This study has some limitations that need to be explained. First, the definition of NK cell subsets is based on positivity or negativity of expression of these markers, instead of evaluating the intensity of expression of said markers. However, if we extrapolate our results to the populations described in the literature, we might fathom that the development of advanced stages of liver fibrosis is related to an alteration of the NK cell maturation process [11, 33, 36]. Second, even though it has been proposed the determination of NKT cells by the coexpression of T cell specific markers, such as CD3, together with NK cell specific markers, such as CD56 or CD16, it is most commonly used the determination of the CD1d protein through analogues of its ligand, α-galactosylceramide [8, 35, 36].

In conclusion, in heavy drinker with advanced fibrosis there are a greater number of total NK cells with an increased cytotoxic phenotype and decreased cytokine-secreting phenotype and expansion of activated T cells. These characteristics could have opposite effects on the progression of liver disease. The expansion of activated T cells could be a compensatory mechanism to maintain the inflammatory context preventing NK cell-triggered cytotoxicity. Deepening the interactions between cell populations in different stages of liver disease could detail the pathophysiology of the disease.

## References

[1] Lackner C, Tiniakos D (2019). Fibrosis and alcohol-related liver disease. J Hepatol.

[2] Higashi T, Friedman S, Hoshida Y (2017). Hepatic stellate cells as key target in liver fibrosis. Adv Drug Deliv Rev.

[3] Fasbender F, Widera A, Hengstler J, Watzl C (2016). Natural killer cells and liver fibrosis. Front Immunol.

[4] Spengler U, Nischalke D, Nattermann J, Strassburg P (2013). Between Scylla and Charybdis: the role of the human immune system in the pathogenesis of hepatitis C. World J Gastroenterol: WJG.

[5] Radaeva S, Sun R, Jaruga B, Nguyen V, Tian Z, Gao B (2006). Natural killer cells ameliorate liver fibrosis by killing activated stellate cells in NKG2D-dependent and tumor necrosis factor-related apoptosis-inducing ligand-dependent manners. Gastroenterology.

[6] Jiao G, Wang B. NK cell subtypes as regulators of autoimmune liver disease. Gastroenterol Res Pract. 2016;2016:6903496. 10.1155/2016/6903496 PMC4947642 27462349

[7] Langhans B, Alwan A, Kramer B (2015). Regulatory CD4+ T cells modulate the interaction between NK cells and hepatic stellate cells by acting on either cell type. J Hepatol.

[8] Balato A, Unutmaz D, Gaspari A (2009). Natural killer T cells: an unconventional T-cell subset with diverse effector and regulatory functions. J Invest Dermatol.

[9] Ochi M, Ohdan H, Mitsuta H (2004). Liver NK cells expressing TRAIL are toxic against self hepatocytes in mice. Hepatology.

[10] Oras A, Quirant-Sanchez B, Popadic D (2020). Comprehensive flow cytometric reference intervals of leukocyte subsets from six study centers across Europe. Clin Exp Immunol.

[11] Michel T, Poli A, Cuapio A (2016). Human CD56bright NK cells: an update. J Immunol.

[12] Amand M, Iserentant G, Poli A (2017). Human CD56 dim CD16 dim cells as an individualized natural killer cell subset. Front Immunol.

[13] Hudspeth K, Donadon M, Cimino M (2016). Human liver-resident CD56(bright)/CD16(neg) NK cells are retained within hepatic sinusoids via the engagement of CCR5 and CXCR6 pathways. J Autoimmun.

[14] Moroso V, Metselaar H, Mancham S (2010). Liver grafts contain a unique subset of natural killer cells that are transferred into the recipient after liver transplantation. Liver Transpl.

[15] Kremer M, Hines N (2008). Natural killer T cells and non-alcoholic fatty liver disease: fat chews on the immune system. World J Gastroenterol: WJG.

[16] Maricic I, Sheng H, Marrero I (2015). Inhibition of type I NKT cells by retinoids or following sulfatide-mediated activation of type II NKT cells attenuates alcoholic liver disease. Hepatology.

[17] Krijgsman D, de Vries L, N, Skovbo A, (2019). Characterization of circulating T-, NK-, and NKT cell subsets in patients with colorectal cancer: the peripheral blood immune cell profile. Cancer Immunol Immunother.

[18] Gao B, Radaeva S (2013). Natural killer and natural killer T cells in liver fibrosis. Biochim Biophys Acta.

[19] Wang L, Jiang W, Wang X, Tong L, Song Y (2022). Regulatory T cells in inflammation and resolution of acute lung injury. Clin Respir J.

[20] Langhans B, Kramer B, Louis M (2013). Intrahepatic IL-8 producing Foxp3^+^CD4^+^ regulatory T cells and fibrogenesis in chronic hepatitis C. J Hepatol.

[21] Zuluaga P, Sanvisens A, Martínez-Cáceres E (2017). Over-expression of CD8 + T-cell activation is associated with decreased CD4 + cells in patients seeking treatment of Alcohol Use Disorder. Drug Alcohol Depend.

[22] Zhou Y, Zhang H, Yao Y (2022). CD4+ T cell activation and inflammation in NASH-related fibrosis. Front Immunol.

[23] Li S, Tan H, Wang N (2019). Recent insights into the role of immune cells in alcoholic liver disease. Front Immunol.

[24] American Psychiatric Association (2022). Diagnostic and statistical manual of mental disorders. Diagn Stat Man Ment Disord.

[25] Rasmussen D, Thiele M, Johansen S (2021). Prognostic performance of 7 biomarkers compared to liver biopsy in early alcohol-related liver disease. J Hepatol.

[26] Zuluaga P, Teniente-Serra A, Fuster D (2022). Increased natural killer cells are associated with alcohol liver fibrosis and with T cell and cytotoxic subpopulations change. J Clin Med.

[27] Ali S, Shahab S, Rauf M (2022). Neutrophil to lymphocyte ratio as a predictor of severity in colorectal adenocarcinoma. J Ayub Med Coll Abbottabad.

[28] Sui Q, Zhang X, Chen C (2022). Inflammation promotes resistance to immune checkpoint inhibitors in high microsatellite instability colorectal cancer. Nat Commun.

[29] Li L, Zhang H, Feng G (2022). Neutrophil-to-lymphocyte ratio predicts in-hospital mortality in intracerebral hemorrhage. J Stroke Cerebrovasc Dis.

[30] Kim T, Park S, Ko S (2022). Dynamic change of neutrophil-to-lymphocyte ratio and symptomatic intracerebral hemorrhage after endovascular recanalization therapy. J Stroke Cerebrovasc Dis.

[31] Lin C, Li Y, Wang Y, Chang W (2022). Higher neutrophil-to-lymphocyte ratio was associated with increased risk of chronic kidney disease in overweight/obese but not normal-weight individuals. Int J Environ Res Public Health.

[32] Jeong W, Park O, Gao B (2008). Abrogation of the antifibrotic effects of natural killer cells/interferon-gamma contributes to alcohol acceleration of liver fibrosis. Gastroenterology.

[33] Cichocki F, Grzywacz B, Miller J (2019). Human NK Cell Development: One Road or Many?. Front Immunol.

[34] Zuluaga P, Sanvisens A, Teniente A (2016). Wide array of T-cell subpopulation alterations in patients with alcohol use disorders. Drug Alcohol Depend.

[35] Liechti T, Roederer M (2019). OMIP-058: 30-Parameter Flow Cytometry Panel to Characterize iNKT, NK, Unconventional and Conventional T Cells. Cytometry A.

[36] Le Nours J, Praveena T, Pellicci D (2016). Atypical natural killer T-cell receptor recognition of CD1d–lipid antigens. Nat Commun.

